# Language Evolution in Swarm Robotics: A Perspective

**DOI:** 10.3389/frobt.2020.00012

**Published:** 2020-02-11

**Authors:** Nicolas Cambier, Roman Miletitch, Vincent Frémont, Marco Dorigo, Eliseo Ferrante, Vito Trianni

**Affiliations:** ^1^School of Computing, University of Leeds, Leeds, United Kingdom; ^2^IRIDIA, Université Libre de Bruxelles, Brussels, Belgium; ^3^UMR CNRS 6004 LS2N, Ecole Centrale de Nantes, Nantes, France; ^4^Computational Intelligence Group, Vrije Universiteit Amsterdam, Amsterdam, Netherlands; ^5^Institute of Cognitive Sciences and Technologies, Italian National Research Council, Rome, Italy

**Keywords:** swarm robotics, language evolution, language games, self-organization, cultural evolution, communication

## Abstract

While direct local communication is very important for the organization of robot swarms, so far it has mostly been used for relatively simple tasks such as signaling robots preferences or states. Inspired by the emergence of meaning found in natural languages, more complex communication skills could allow robot swarms to tackle novel situations in ways that may not be a priori obvious to the experimenter. This would pave the way for the design of robot swarms with higher autonomy and adaptivity. The state of the art regarding the emergence of communication for robot swarms has mostly focused on offline evolutionary approaches, which showed that signaling and communication can emerge spontaneously even when not explicitly promoted. However, these approaches do not lead to complex, language-like communication skills, and signals are tightly linked to environmental and/or sensory-motor states that are specific to the task for which communication was evolved. To move beyond current practice, we advocate an approach to emergent communication in robot swarms based on language games. Thanks to language games, previous studies showed that cultural self-organization—rather than biological evolution—can be responsible for the complexity and expressive power of language. We suggest that swarm robotics can be an ideal test-bed to advance research on the emergence of language-like communication. The latter can be key to provide robot swarms with additional skills to support self-organization and adaptivity, enabling the design of more complex collective behaviors.

## 1. Introduction

Swarm robotics is an approach to the study of multi-robot systems that aims at designing complex collective behaviors by means of relatively simple robots. A key factor in the design of robot swarms is self-organization, which results from the numerous local interactions among robots and between robots and their environment (Şahin, 2004; Brambilla et al., 2013). Similarly to other features of robot swarms, such local interactions are often designed after natural systems, animal societies being the most relevant source of inspiration.

Research in swarm robotics generally considers three forms of interactions: indirect communication, direct interactions, and direct communication (Trianni and Dorigo, 2006). The first, mostly present in insect societies, is often referred to as *stigmergy*, which is a form of indirect communication that takes place through modifications of the environment, as for instance in ants depositing pheromones during their foraging trips (Deneubourg et al., 1990; Garnier et al., 2007). Direct interactions entail some influence among individuals through physical contact, which provokes a response on the receiver (e.g., pulling/pushing forces during collective transport; Kube and Bonabeau, 2000). The third form of interaction is direct communication, which implies the concurrent exchange of information among individuals without physical contact, and it is by far the most ubiquitous modality of interaction in swarm robotics. Direct communication is implementable with radio links, simple devices like infra-red transceivers or through visual signals (e.g., colored LEDs), whereas indirect communication and direct interactions require much more specific sensors and actuators [e.g., UV-light emitters coupled with a bespoke floor material (Alers et al., 2014) or force/torque sensors (Groß et al., 2006)]. Direct communication has been used for diverse tasks such as self-organized aggregation (Soysal and Sahin, 2005), morphogenesis (O'Grady et al., 2009), foraging (Ducatelle et al., 2011b), or flocking (Ferrante et al., 2014), and it also proved useful to emulate other forms of interaction (e.g., robot chains to emulate pheromones) (Nouyan et al., 2009; Campo et al., 2010; Ducatelle et al., 2011a; Ferrante et al., 2013).

In the cases presented above, the rules of communication are designed specifically for the task at hand, offering little to no adaptation toward variations of the task or changes in the working environment. This creates a clear limitation to swarms' autonomy. Consider, for example, the case of self-assembly and coordinated motion, which have been implemented in robot swarms to negotiate obstacles such as hills or holes during exploration of complex unstructured environments (O'Grady et al., 2010). If the conditions necessary to trigger the self-assembly in a given shape are predefined by the experimenter and are specific for a few types of obstacles, no adaptation is possible when the robots have to navigate in an heterogeneous environment with obstacles of different kinds. Therefore, for robot swarms to cope with uncertain environments with unknown obstacles, the set of shapes in which they can self-assemble should be part of the robots' action and communication space. Conversely, the rules of communication should be sufficiently adaptable to induce the type of self-assembly required for said obstacles. This adaptability is even more important when the nature of the tasks itself might change over time.

In this perspective, we propose that the adaptivity necessary to tackle unpredictable environments should be addressed through self-organization of the communication process. A richer communication ability should make swarms capable of advanced negotiation dynamics, leading to the autonomous and self-organized formation of different categories for contexts that require different responses. As we will see in section 2, steps have already been made in emerging communication systems adapted to different environmental contexts, but these studies do not propose post-deployment adaptivity. A possible mechanism to achieve online adaptivity that we propose here is represented by language games (LG), introduced in section 3. LGs share with swarm robotics an emphasis on local interactions among agents and on self-organization within a population. Section 4 will show how these similarities make language games easy to combine with swarm robotics behaviors, also illustrating some examples of current research. Finally, section 5 discusses the advantages and limitations of the approach proposed in this paper.

## 2. First Steps in the Evolution of Communication

Except for a subset of studies that use artificial systems as a means to explain linguistic features (see section 3), efforts in evolving communication for multi-robot systems have mostly focused on automatic design (Nolfi and Mirolli, 2009), often within the framework of evolutionary robotics (Nolfi and Floreano, 2000). The main challenge in evolving a functional communication system stands in the need to concurrently determine both the signal and a suitable response to the signal. Each of the two traits, if taken individually, may be either maladaptive or neutral and can easily be selected out by the automatic design process. Hence, specific conditions must be met to observe the emergence of communication.

The situation grows in complexity when a non-trivial swarm behavior must be synthesized. As a matter of fact, only a fraction of the research on evolving communication is useful for robot swarms. In a prominent study on this topic, small colonies of robots were evolved within a particular scenario that did not especially encourage communication (Floreano et al., 2007). In this experiment, the robots were assigned a foraging task (i.e., find a food source in order to feed). However, the environment also hosted poison sources indistinguishable from food sources. At the end of an evolutionary optimization process, the robot swarms equipped with a visual communication system showed significantly better performance with respect to communication-less swarms. Specifically, two types of signals emerged in different populations, whereby agents either shared the position of the food sources to attract teammates, or they signaled the poison sources to repel other robots.

Other works used evolutionary robotics to evolve signaling for categorization of environmental features (Ampatzis et al., 2008), expecting these signaling systems to produce more adaptable behaviors (according to the rationale we presented in section 1), especially when porting controllers evolved *in silico* to the real world. In successfully evolved controllers, signaling emerged without any incentive as a cue to distinguish between two different environments, that the robots could recognize only after some exploration. Minimal instances of communications have also been evolved to allow the synchronization of a swarm (Trianni and Nolfi, 2009), as well as to coordinate the activities in robot pairs (Tuci, 2009; De Greeff and Nolfi, 2010; Uno et al., 2011). Besides evolutionary approaches, other automatic design methods have been proposed that are capable of producing efficient communication for behaviors such as aggregation, coordination, and categorization (Hasselmann et al., 2018).

By looking at these and similar research studies not mentioned here for brevity, some important lessons can be learned. Indeed, communication systems may emerge spontaneously, even if there is no explicit reward (e.g., no selective pressure from the fitness function in the evolutionary robotics approach). Furthermore, they evolve to provide an advantage to evolved populations compared to those that evolved without means to communicate.

Except for a handful of examples that present features of communication necessary for the emergence of human languages (i.e., compositionality and joint attention) (Tuci, 2009; Uno et al., 2011), the studies available in the swarm robotics literature obtained communication systems limited to simple instances of signaling, very far away from the complex communication schemes that characterize animal societies and, of course, humans. Indeed, the emerged communication systems are defined as a limited set of fixed signals triggering a pre-determined and hard-wired reaction in conspecifics, akin to early definitions of signaling (Owren et al., 2010). This is especially obvious in several studies within the swarm robotics literature (Trianni and Dorigo, 2006; Ampatzis et al., 2008; Trianni and Nolfi, 2009; Tuci, 2009), wherein the signal produced by an individual robot yields reactions from its neighbors as well as from itself.

Automatic design methods present several disadvantages for the evolution of communication. Indeed, these design methods rely on simplistic building blocks [neurons (Trianni, 2008) or behavior modules (Francesca et al., 2014)], allowing for little variety in the resulting communication processes. Moreover, the emergence and evolution of the communication rules is strictly confined to the training step and thus the evolved rules remain identical after deployment. The current use of automatic design methods therefore limits the adaptivity necessary for communication in uncertain environments.

## 3. Language Games

Multi-agent artificial systems have been widely used to study the evolution of natural languages *in silico*. The seminal “talking heads” (Steels, 2015) experiment was the first to showcase self-organization of language in a complex system within a population of artificial agents.

This approach does not rely on evolutionary computation or any other automatic design methods to grow the language mechanics. Instead, inspiration is from linguistics, whereby the self-organization of natural languages is classified as cultural evolution, in opposition to biological evolution. Cultural evolution is the result of the sum of local interactions based on confirmation and agreement rules. In computer science, such interactions are often modeled by simple games played by a population of agents, seen as potential ways for them to cooperate (Ackley and Littman, 1994). When linguistic interactions are considered, a *language game* can be defined to be played between agents/robots, turn by turn, with the purpose of mimicking real-world dynamics leading to the emergence of a structured language. Language games make direct reference to concepts developed in the philosophy of language (Wittgenstein, 1953). If, according to Wittgenstein, language games were simple abstractions of a real-world language, in computer science language games are minimal algorithms that display, in an artificial context, the salient characteristics of a whole language.

Various kinds of language games have been proposed to date, such as the imitation game which, in De Boer (2000), deals with vowel vocalization. In this work, agents are equipped with an articulatory synthesizer, a module for calculating the distances between different vowels (according to human perception) and a repertoire for storing vowel prototypes. Then, two agents (among many) are selected randomly and start the game interaction. The first agent (*the initiator*) selects a random vowel from its repertoire and utters it. The second agent (*the imitator*) then tries to imitate this vowel by uttering the closest vowel in its own repertoire. The initiator subsequently has to find the closest vowel to the one uttered by the imitator in its own repertoire, the goal being to thus find the initial vowel. Depending on the issue of previous games and on the success of the current one, both agents then either “merge” their vowels (they shift their vowel in the articulatory space toward the one they perceived) or add a new one. This protocol, coupled with some communication noise, causes the emergence of vowel systems that are strikingly similar to those found in actual human languages because the agents self-organize in order to produce vowels that are as distinguishable from each other as possible.

The imitation game requires to separate the agents into initiator and imitator categories. Other language games instead rely on a speaker and a hearer, which are interchangeable roles for the agents playing the game. This is the case for the *guessing game* (Steels, 2001), where the speaker chooses a concept within a context (physical or abstract), and communicates the corresponding word to the hearer. The latter has to guess which topic was chosen based on the communicated word. If it fails, both speaker and hearer update their inner representation of the concept. The guessing game can be seen as an implementation of the *Gavagai* thought experiment (Quine, 2013), addressing the inscrutability of reference in a computational context, that is, the fact that one word can never have exactly the same meaning for different agents. Similar to the guessing game, the *category game* (Puglisi et al., 2008; Baronchelli et al., 2010) aims to self-organize discrete sub-intervals of one or many perceptual channels through negotiation dynamics. The agents start without any predefined category, and develop a pattern of categories shared among the agents via repeated interactions. Eventually, a global agreement emerges within the population. The negotiation dynamics lead to a *communication grounding* (as detailed in Clark and Brennan, 1991) amongst all agents, assuring a matching signified/signifier link between words and concepts to be exploited in future communications. The category game can be simplified into the *naming game* (Steels, 1995, 2003) where categories are provided from the beginning, placing the emphasis of the game on the negotiation dynamics and the emergence of an agreement.

## 4. Language Games for Robot Swarms

In this paper, we propose that the exploitation of the compatibility between language games and swarm robotics can yield great results, both in enhancing the efficiency and adaptivity of the communication between the robots in the swarm and in providing new means to study the evolution of language. A key ingredient for both swarm robotics and language evolution is self-organization in a population of agents resulting from local interactions. This is a dynamic process that comes about within the population in response to the contingencies experienced by the agents themselves while displaying their behavior, resulting in a never-ending evolution that can react to changes in the environment, with new concepts/words arising when needed. This contrasts with the evolutionary studies mentioned in section 2, where the communication system was encoded offline by means of an optimization process, without much space for online adaptation.

Various degrees of coupling are possible between language games and swarm robotics. First, at the lowest level of complexity, the robot behavior is not affected at all from the language game, which is simply played by the robots upon repeated encounters. Second, the language game can affect the behavior of the robots, but the latter have no direct influence on the way in which language evolves. Finally, in the third case, the behavior of the robot affects the evolving language. These three couplings are considered in the studies presented below. The higher the coupling between the language and the robot behavior (the second and third cases can be mixed), the higher the complexity of the emergent swarm behavior, as the swarm is allowed to exploit the full spectrum of the language complexity (Five Graces Group et al., 2009). As will become clear with the examples, in the first two cases, the language only carries information useful for the observer (i.e., it provides descriptors of the environment/task). However, in the latter case, the language carries an emergent semantics that is actually intrinsic to the system, i.e., language can be purposely used by the robots themselves. This is a necessary feature for developing grounded symbols (Harnad, 1990), as we will discuss in section 5.

**Figure 1:**
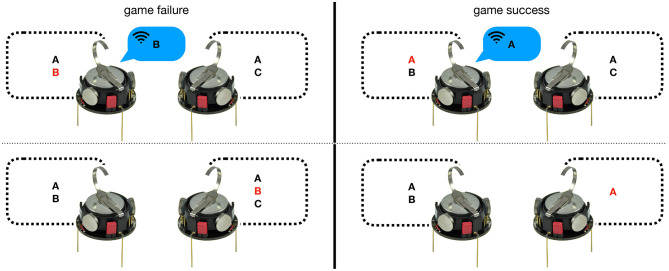
Interaction between two Kilobot robots (Rubenstein et al., 2014) playing the MNG, with both possible outcomes: success and failure. In both cases, the left robot (i.e., the speaker) chooses one of the words from its inventory and broadcasts it to all the other robots in range. The right robot (i.e., the hearer) checks the existence of the word in its inventory and updates it. In case of game failure **(left)**, the word is included into the inventory. In case of game success **(right)**, the word is maintained in the inventory and all others are excluded.

Currently, a few attempts have been made to exploit the power of language games for the coordination of robot swarms. One language game in particular has received a lot of attention: the *minimal naming game* (MNG, see Steels, 1995, 2003; Baronchelli et al., 2006; Baronchelli, 2016). In this game, two or more robots interact to assign a unique name to a single object, similarly to the imitation game, as illustrated in Figure 1.

Here, focus is given to reaching consensus on a single word for a single object within a population of communicating robots. Trianni et al. (2016) studied the consensus dynamics generated by the MNG in a dynamic network formed by robots moving about in a bounded arena, without any interaction between language game and robot behavior. In this research, the communication network was shaped by the encounters between the robots, each independently performing a simple random walk. This work concluded that the collisions between wireless transmitted messages, due to the simple communication protocol and the relatively high density of robots used in the experiments, led to the abortion of a significant portion of games. This turned to be a positive fact as the strain on the robots' memory was thus reduced, which is advantageous considering the limited capacities assumed in swarm robotics (Brambilla et al., 2013). Moreover, the embodiment of the robots and their collisions led to the formation of aggregates of robots that do not easily disband, leading to a reduced interaction rate in the population and a slower convergence with respect to simulated agents. This second phenomenon impacts the capacity of information transfer within the swarm, but does not impair the ability of the swarm to reach consensus, albeit with longer delays.

Building on this, Miletitch et al. (2019) focus on the understanding of possible correlations between a foraging task and the language dynamics resulting from an embodied version of the MNG. In this setup, the topology of the interaction network is determined by the foraging task, introducing a transition in time from a well-mixed population to a swarm polarized in two sub-populations with little contact. In this setup, the creation of the words used for the MNG is anchored to the resource from which robots forage: a robot creates a word when it discovers a resource new to him. In other words, the behavior of the robots influences the language dynamics, but not the other way round. It results that the emerging vocabulary maintains an accurate description of the environment, which is both complete (there is a single word for each existing resource) and correct (no resource is referred to with a name created elsewhere). Such description is maintained for as long as each resource is relevant to the swarm, that is, as long as there are robots foraging from it. Overall, the language game provides a naming system that adapts to the features of the environment as well as to the way in which the task is performed by the robot swarm.

Recent studies focused on the effects that a self-organized behavior and the MNG can have on each other. In Cambier et al. (2017), a swarm of robots performed self-organized aggregation and concurrently played a MNG where the exchanged words were used to identify the aggregate to which robots would belong to. Under specific density conditions, robots split into a controllable quantity of coalitions, each characterized by a different word used as identifier. In Cambier et al. (2018), a further interaction was considered, as the words used within the MNG encode the parameter of the aggregation controller, directly impacting the quality of the self-organized aggregation behavior. As a matter of fact, in the MNG, words supported by highly-connected agents propagate more (Baronchelli, 2011). This means that agents that aggregate better propagate widely their words, and, thus, the aggregation parameters. This creates a positive-feedback loop that selects and maintains parameters promoting the formation of stable and large aggregates. Variations of the available words are introduced in the swarm via errors in the communication, which is modeled as a stochastic process in which some bits are flipped during the transmission. Such variations allow to explore new aggregation parameters, which can lead to changes in the way robots behave. The dynamics of the MNG therefore leads to the cultural evolution of the aggregation behavior itself.

## 5. Discussion

Natural languages are a tool that arose (at least partly) from the need to purposely share information (Noble and Davidson, 1991). As a matter of fact, the rise for a complex communication system is linked to specific tasks to be accomplished, and ultimately, to survival of the population. In this perspective, we proposed that language games and swarm robotics should be combined as both have decentralization and self-organization at their roots. This similar emphasis would allow to produce communication systems that evolve online, that are exploited to represent a dynamic and uncertain environment and that can be used to improve performance in task execution. Self-organizing languages should therefore enable robots to describe and tackle new challenges as they come. They are necessary to build truly autonomous robot swarms.

In addition to their focus on self-organization, language games address several challenges related to the development of complex languages. First is the symbol grounding problem, i.e., how to associate a word to experiences of the real world (Harnad, 1990). Most studies on language games rely on linking perceptions of the environment to names. Indeed, as robots have different bodies, they can experience the environment with different modalities and from different points of view, so that the sensory data they associate with a shared word is usually not exactly the same. This association mechanism only requires simple feature extraction algorithms and a bidirectional memory (mapping between words and meanings, as in Steels and Loetzsch, 2012). The consensus dynamics of language games ensures the symbol grounding, although each robot has its own internal representation of each word's meaning. This can cause issues in communication early on, but can result in a very efficient system once a robust association between concepts and experiences has been established. This favors especially “uneducated” robots that join the swarm later on, as they can quickly acquire new concepts from few interactions with their peers. Nevertheless, alternative combinations where swarms of robots learn to link actions to verbs are possible and could provide interesting new abilities. Some work has already been performed in that direction using language games (Steels, 2008), and additional efforts on symbol grounding could also take advantage from *developmental language acquisition* models (Rasheed and Amin, 2016). Such models use neural networks to incrementally teach agents (in a teacher/learner scenario) new meanings, starting from observable objects before moving onto more abstract concepts or actions.

Relevant studies in the evolution of language have shown that a simple grammar can be generated, exploiting the plasticity of the learned language (Spranger et al., 2010). The emergence of a simple compositionality in the language can lead to more complex expressions, paving the way to full sentences. This is of great relevance for swarm robotics studies in order to address complex tasks that require multiple actions to be scheduled and coordinated among robots. An efficient scheduling and coordination plan can emerge from local knowledge to be shared among robots, without any pre-defined structure or plan. By exploiting the compositionality of language, a sequence of tasks can be defined and eventually executed, leading to the emergence of swarm behaviors that are far more complex than the current state of the art. Through the development of *fluid construction grammars* (Steels and De Beule, 2006), language games can evolve grammatical structures as well as lexicons (Beuls and Steels, 2013). The dynamics of such games have, however, not been as thoroughly studied as, e.g., the naming game, and are not ready for implementation in swarm robotics systems. The robotics community should therefore attempt to facilitate the development of such language games in order to be able to benefit from them.

Whereas, the studies discussed in section 3 focused on language games without an underlying task, in swarm robotics robots are meant to execute some task in an efficient manner. Language can therefore emerge in such context to support the task execution. On one hand, this makes swarm robotics an appropriate test-bed to study language emergence in a physical setting, giving a chance to concurrently develop *language and the actions into which it is woven*, as initially theorized by Wittgenstein (1953). On the other hand, the works presented in section 4 have laid the foundations for an approach that holds the potential to create complex and flexible swarm behaviors. Future work could either aim at using language games other than the naming game in appropriate swarm robotics scenarios, or focus on developing more advanced language games following the framework that has already been developed in current studies.

Furthermore, the work presented in this perspective very much assumes *a priori* the existence of a conversational frame (i.e., the language game protocol) and a sociological frame (i.e., the swarm behavior) (Goffman, 1974). Future work could aim at making these frames emerge as well, perhaps during *a priori* experiments in controlled environments. In this view, it clearly appears that the goal of this perspective is not to diverge from the efforts that have already been made to evolve communication in swarm robotics, but, rather, to propose a new direction for them, which offers post-deployment adaptivity.

Finally, we should keep in mind that our proposed approach also has some drawbacks. Indeed, language games require memory and are therefore non-Markov processes. Therefore, they add a layer of complexity to swarm systems, which are already complex and difficult to predict. We believe that this additional layer of complexity is necessary for scenarios in uncertain environments and where the task can change over time. However, it is necessary to concurrently develop methods and tools for the analysis of swarm behaviors that are affected by an evolving language.

## Author Contributions

NC, RM, EF, and VT initially conceived the paper. All authors contributed to the article preparation, writing, and revision.

### Conflict of Interest

The authors declare that the research was conducted in the absence of any commercial or financial relationships that could be construed as a potential conflict of interest.
